# Connecting intercity mobility with urban welfare

**DOI:** 10.1093/pnasnexus/pgac178

**Published:** 2022-09-07

**Authors:** Sayat Mimar, David Soriano-Paños, Alec Kirkley, Hugo Barbosa, Adam Sadilek, Alex Arenas, Jesus Gómez-Gardeñes, Gourab Ghoshal

**Affiliations:** Department of Physics & Astronomy, University of Rochester, Rochester, NY 14607, USA; Instituto Gulbenkian de Ciência, 2780-156 Oeiras, Portugal; GOTHAM Lab Department of Condensed Matter Physics and Institute for Biocomputation and Physics of Complex Systems (BIFI), University of Zaragoza, E-50009 Zaragoza, Spain; Institute of Data Science, University of Hong Kong, 999077, Hong Kong; Department of Urban Planning and Design, University of Hong Kong, 999077, Hong Kong; Urban Systems Institute, University of Hong Kong, 999077, Hong Kong; Department of Computer Science, University of Exeter, North Park Road, Exeter EX4 4QF, UK; Google Inc., 1600 Amphitheatre Parkway, Mountain View, CA 94043, USA; Department d’Enginyeria Informática i Matemàtiques, Universitat Rovira i Virgili, Tarragona 43007, Spain; GOTHAM Lab Department of Condensed Matter Physics and Institute for Biocomputation and Physics of Complex Systems (BIFI), University of Zaragoza, E-50009 Zaragoza, Spain; Department of Physics & Astronomy, University of Rochester, Rochester, NY 14607, USA

## Abstract

While significant effort has been devoted to understand the role of intraurban characteristics on sustainability and growth, much remains to be understood about the effect of interurban interactions and the role cities have in determining each other’s urban welfare. Here we consider a global mobility network of population flows between cities as a proxy for the communication between these regions, and analyze how it correlates with socioeconomic indicators. We use several measures of centrality to rank cities according to their importance in the mobility network, finding PageRank to be the most effective measure for reflecting these prosperity indicators. Our analysis reveals that the characterization of the welfare of cities based on mobility information hinges on their corresponding development stage. Namely, while network-based predictions of welfare correlate well with economic indicators in mature cities, for developing urban areas additional information about the prosperity of their mobility neighborhood is needed. We develop a simple generative model for the allocation of population flows out of a city that balances the costs and benefits of interaction with other cities that are successful, finding that it provides a strong fit to the flows observed in the global mobility network and highlights the differences in flow patterns between developed and developing urban regions. Our results hint towards the importance of leveraging interurban connections in service of urban development and welfare.

Significance Statement Determining the factors behind the economics success of urban areas is a complex endeavor involving many factors. Nevertheless, the flow of intellectual capital and resources between them likely plays a role. Here we analyze the global mobility network of cities, finding that their position in the network, combined with their topological properties are good predictors of their success. Developing cities leverage their mobility neighborhood, connecting to developed cities to rise up the success ladder.

## Introduction

Given the recent trend of rapid urbanization wherein the majority of the global population now resides in urban centers (1,2), cities are at the center of innovation and technological advancement (3,4). The current relevance of cities has fueled the birth of the so-called *science of cities* aimed at uncovering the physical and structural features driving their growth and function (5–7). Among the different topics addressed by this discipline, one recurrent question has been that of quantifying how human mobility shapes urban dynamics and provides reliable information on different socioeconomic indicators. Along this line, recent studies have used intracity mobility flows to understand spatial city organization and its connection to urban characteristics such as facilities, jobs, and services that are crucial for city livability and sustainability (8,9). Other works have investigated accessibility in urban systems considering city topology and infrastructure that mediate interactions and activities of inhabitants (10–12). More generally, mobility flows have been used to study the structure of urban areas and dynamics taking place within them (13–17).

The welfare of a city is just one example of the emergence of success within a system composed of a large set of similar elements among which there are relations of competition and cooperation. The mechanisms driving the emergence of success have been recently investigated in a variety of fields; for instance, luck and randomness have been found to be crucial features behind the impact and productivity of scientific (18, 19), or creative careers (20). Apart from the impact of intrinsically stochastic events, the roots of individual success in different disciplines has been addressed through the lens of network science. Specifically, in (21), the authors capture institutional prestige by studying a co-exhibition network where nodes represent art galleries and the links are determined by the movement of artists among institutions. Similarly, in (22), the network of professional relationships of start-ups is built with the flows of employees spanning over 26 years of professional relationships among world-wide companies. This network-based approach has shown to have predictive power of the future success of companies. Finally, in (23), the authors construct a workforce mobility network among metropolitan areas in the United States and predict diverse socioeconomic outputs of urban areas such as number of new patents, the number of R$\&$D establishments, and total wages.

For the case of urban system, there are multiple factors shaping their success; common elements of urban growth that have been considered are local economic policies (24–26), immigration (27,28), pool of skilled labor (29), and luck (30). A recent study argues economic specialization, human capital formation, and institutions (31–33) as essential components of economic development for urban areas (34). Moreover, several network-based approaches have been proposed to uncover mechanisms behind urban growth at local scale (35–37). In (38), authors study the extent of the regional development levels by adopting a network representation: regional economies are embedded in networks of relationships with other economic factors across different spatial scales ranging from local to global (characterizing interfirm, intraorganizational, and community-based interactions), through which growth is stimulated and transmitted (39). These economic relations generate knowledge, access to technologies, resources and markets, and catalyze income growth at regional level (36). Little attention is devoted, however, to understanding the connection of metropolitan development and economic benefit to intercity population flows (40–42). In (43), authors identify the link between air traffic and local economic growth of urban systems by exploiting data of metropolitan areas over a two-decade period and find that air traffic growth rate generates an additional stream of income.

Here we present a large scale analysis at global level that aims at quantifying the connection between human mobility and the socioeconomic development of cities. To this end, we propose a network science approach to shed light on the heterogeneous distribution of success, as measured by various socioeconomic indicators observed among cities worldwide. We use anonymous and aggregated intercity flows to construct a global mobility network between cities, with the aim of establishing a connection between their success with the observed flow patterns. This mobility data includes both local and international travels of populations among urban areas that facilitate business trips and combines several underlying channels that people interact: knowledge and capital flows (44), labor mobility (45), and foreign direct investment (FDI) trade (46,47). For these forces that incorporate and trigger development, long range contacts, face-to-face meetings, and exchange with collaborators in other cities are crucial to build economic relationships and promote productivity (40, 48).

We start by ranking cities with respect to various network centrality measures—in particular connectivity, PageRank, and eigenvector centrality—which capture not only the strength of connections but also their quality, and compare these centralities with socioeconomic indicators in 268 metropolitan areas worldwide. We find that information provided by network centrality measures is strongly correlated with metrics of welfare in well-developed cities, whereas it is much more weakly associated with those corresponding to developing areas. For the latter, we demonstrate that welfare is strongly connected to the welfare of their neighborhood, that is, those cities with which it has the highest volume of interurban population flows. Finally, we build a simple model for the outgoing flows from a city, finding that the prosperity of a city is a major element in attracting flows from others, and that less developed cities place greater priority on directing flows to highly successful cities.

## Results

### Data description

#### Mobility data

The Google Aggregated Mobility Research Dataset contains anonymized mobility flows aggregated over users who have turned on the Location History setting, which is off by default. This is similar to the data used to show how busy certain types of places are in Google Maps, which helps to identify when a local business tends to be the most crowded. The flows are between cells of approximately 5 km^2^ for the year 2019 and is aggregated weekly.

To produce this dataset, machine learning methods are applied to logs data to automatically segment it into semantic trips (8). To provide strong privacy guarantees, all trips are anonymized and aggregated using a differentially private mechanism (https://research.google/pubs/pub48778/) to aggregate flows over time (https://policies.google.com/technologies/anonymization). No individual user data were ever manually inspected, only heavily aggregated flows of large populations were handled.

All anonymized trips are processed in aggregate to extract their origin and destination location and time. For example, if *n* unique users traveled from location *a* to location *b* within week *w*, the corresponding cell (*a, b,w*) in the mobility tensor would have a value of *n* ± η, where η is noise drawn from a Laplace distribution with mean 0 and scale 1/0.66. All metrics are removed for which the noisy number of users is lower than 100. This automated Laplace mechanism yields a (*ϵ, δ*)-differential privacy guarantee of *ϵ* = 0.66 and *δ* =2.1 × 10^−29^ per metric. The parameter *ϵ* controls the noise intensity in terms of its variance, while *δ* represents the deviation from pure *ϵ*-privacy. The closer each value is to zero, the stronger the privacy guarantees. All data processing is done before being made available to researchers, and the analysis in this paper is done on the resulting heavily aggregated and differentially private data.

The mobility flows are encoded in an origin–destination matrix ***T*** whose elements *T_ij_* represent the flows from location *i* to *j* and whose diagonal elements correspond to flows within cells on a weekly basis. For the purposes of our analysis, we aggregate the flows to the full year, that is *T_ij_* corresponds to the total flow between locations *i, j* over the year 2019. We note that the dataset excludes mobility information from China.

#### Socioeconomic indicators

To quantify the economic success of cities, we collected data of city-level socioeconomic variables from the company Jones Lang LaSalle IP, Inc. (JLL) for the year 2018 (51). The report published by JLL gives the gross domestic product (GDP), total real estate investment (TREI), and cross-border real estate investment (CBREI), and commercial attraction index (BHI) for 300 cities worldwide. In particular, the BHI is a composite measure that accounts for key real estate indicators (investment volumes and commercial real estate stock), as well as socio-economic and business indicators such as economic output, population, corporate presence, and air connectivity (https://seoulsolution.kr/sites/default/files/gettoknowus/jll-global300-2015.pdf).

Besides these continuous variables, there are categorical variables in the dataset that divide cities into different subsets based on their level of development and geographical region. The indicated levels of development (in increasing order) are *Early Growth, Developing, Transitional*, and *Mature*, which are assigned following information concerning the city’s real estate liquidity, the depth of its corporate occupier base, and the quality and range of its commercial stock. The geographical sub-regions indicated are *North America, LATAM $\&$ Caribbean, Asia, Australasia, Western Europe, CEE/CIS, MENA*, and *Sub-Saharan Africa*. For our analysis, we consider 268 metropolitan areas, excluding China, for which we do not have mobility data. (See Figs. S1 and S2, and Table S1 for more details about the urban areas used in this study.)

#### City boundaries

The Organization for Economic Cooperation and Development (OECD) provides boundaries for functional urban areas in member countries (http://www.oecd.org/cfe/regional-policy/functionalurbanareasbycountry.html). Using a gridded population dataset, urban cores are defined as clusters of adjoining grid cells with a population density above a certain threshold—1,500 inhabitants per km^2^ for all regions except Mexico and the United States, where due to lower density the threshold is 1,000 inhabitants per km^2^. Many Asian and African cities are not part of the dataset. For these, we use data from the Atlas of Urban Expansion (AOUE; http://www.atlasofurbanexpansion.org/data), which provides a definition of city boundaries based on the extension of the built-up area.

### Relating welfare and PageRank centrality

While we have metadata for 268 cities, for the purposes of our analysis, we construct the static mobility network of aggregated intercity flows. The network consists of *N* = 1,774 nodes representing global metropolitan areas in our dataset that have a population *P* > 100,000 (https://www.naturalearthdata.com/) and *E* = 39,868 directed edges. To incorporate, in a tunable way, the contribution of long-distance travel to economic activity, we define the edge weights of the mobility network as

**Fig. 1.**
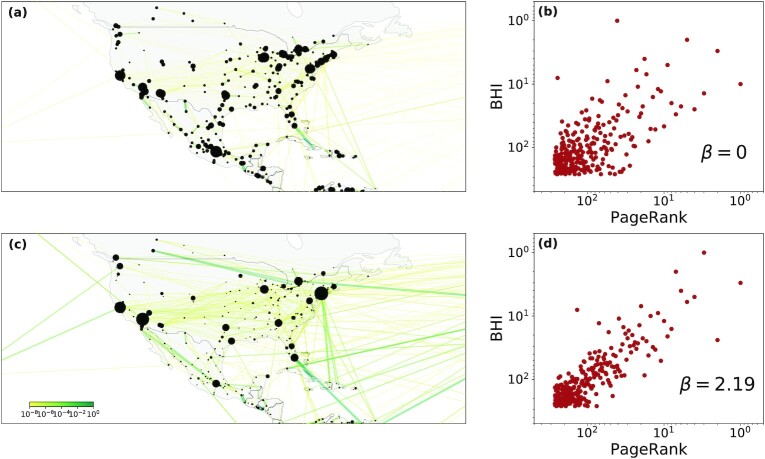
Connecting network position and welfare. Weights *W_ij_*, of the global mobility network for (a) the raw mobility flows (*β* = 0 in Eq. 1) and (**c**) when incorporating the effect of distance in the weights (*β* = 2.19), shown for North American cities. Node sizes are proportional to their PageRank in both maps. By including distance in the flows, we give stronger weight to long-range trips. (b) BHI vs PageRank for *β* = 0, where the horizontal axis indicates the ordered ranking based on their PageRank values, with a Spearman correlation coefficient ρ_*s*_ = 0.53. (d) The same for BHI vs PageRank for *β* = 2.19, yielding ρ_*s*_ = 0.77.

(1) \begin{equation*} W_{ij} = T_{ij} \times d_{ij}^{\beta }, \end{equation*}

where *β* ≥ 0. In this expression, *T_ij_* is the total flow from city *i* to *j* and the *d_ij_* is the geodesic distance between them. Incorporating this allows for including the fact that long-range connections indicate greater levels of implied connectivity between the cities, given the associated cost of travel (56, 57). The optimal value of the exponent *β* is determined later. The effect of distance on the flow-weights is illustrated in Fig. 1 where panel (a) shows mobility flows only (*β* = 0), whereas in panel (b) we show an illustrative example for *β* > 0. As the figure shows, incorporating the effect of distance makes long-range flows more prominent where colors indicate magnitude of flow, increasing from yellow to red, and node sizes correspond to PageRank [maps generated using the Shapely (https://pypi.org/project/Shapely/) and GeoPandas (https://geopandas.org/) packages in Python].

Next we rank cities according to their importance based on their position in the mobility network. An ideal metric for that is the PageRank centrality, a network-based diffusion algorithm, used by Google and other web search engines to estimate the importance or quality of web pages. The algorithm takes into account both the number of connections as well as the importance of the neighbors a node connects to (60,61). For a weighted directed network, the PageRank of a node *i* is computed as (62)

(2) \begin{equation*} PR_{i}=\frac{(1-\alpha )}{N}+\alpha \sum _{j} \frac{{W}_{ji}PR_{j} }{ s^{\mathrm{out}}_j}, \end{equation*}

where $s^{\mathrm{out}}_j$ is the total strength of outgoing links from node *j* and α is a “reset” parameter in the range [0–1] (where we set *α* = 0.85 (60)). In addition to web-pages, the metric has been successfully employed to rank scientists based on their citation patterns (63,64), disease-causing genes based on protein–protein interactions (65), roads or streets in terms of traffic (66), ecological species based on their position in the food web (67), and highlight cancer genes in proteomic data (68) among other applications.

We start our analysis by ranking cities in the mobility network by their value of PageRank and plot it against one of the composite socioeconomic indicators, the BHI. The strength of association is measured by Spearman’s correlation coefficient ρ_*s*_ that measures the strength and direction of association between two ranked variables. In Fig. 1(c) we plot the case for *β* = 0 finding a monotonic relationship with ρ_*s*_ = 0.53. Including distance in the edge-weights reveals an increase in the association between the two variables for any non-zero *β*. In Fig. S3 panels (a) and (b), we plot ρ_*s*_ as well as Pearson’s correlation coefficient ρ_*p*_ between log (BHI) and log (*PR*) as a function of *β*, finding a peak value for both correlation coefficients at *β* = 2.19 (ρ_*s*_) and *β* = 1.88 (ρ_*p*_). In Fig. 1 panel (d), we plot the scatter plot for PageRank and BHI for *β* = 2.19, indicating a much stronger correlation (ρ_*s*_ = 0.77). In Fig. S4, we show the connection of PageRank with other socioeconomic indicators (total real estate investment, GDP, and cross-border real estate investment) for a variety of choices of the edge-weights: the raw flow *T_ij_*, the distance *d_ij_* as well as Eq. (1). The results indicate that (i) PageRank is correlated with all socioeconomic measures (the strongest being BHI), and (ii) this association is enhanced when both the flow and distance is incorporated in the edge-weights. We also show the results for two other centrality measures (weighted degree and eigenvector centrality). Taken together, it appears that both the position of a city in the global mobility network, as well as the strength and number of connections is a reasonable predictor of its welfare.

**Fig. 2.**
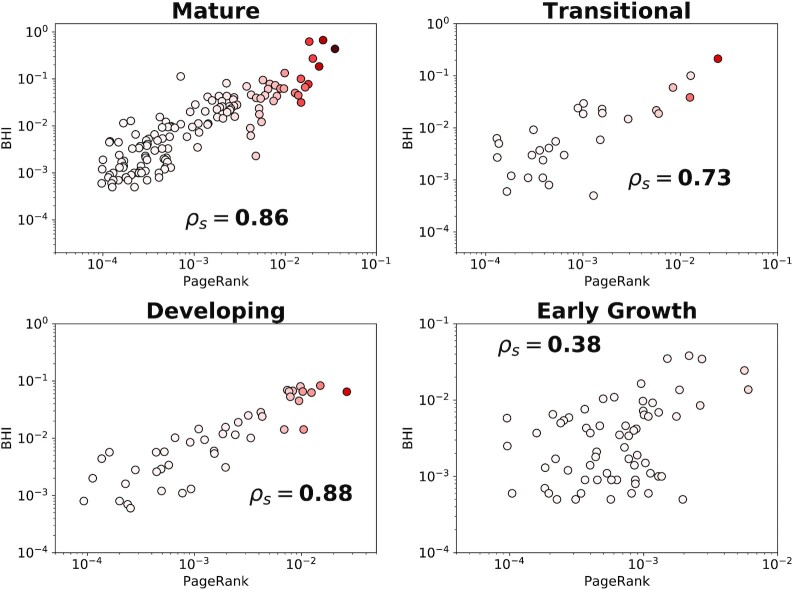
Disaggregating cities by development level PageRank vs BHI when cities are grouped with respect to their development level. Spearman’s correlation in each panel shows association level of the socioeconomic indicator and the network centrality measure. Colors represent the strength of connectivity for each city with dark red indicating higher levels of weighted-degree.

**Fig. 3.**
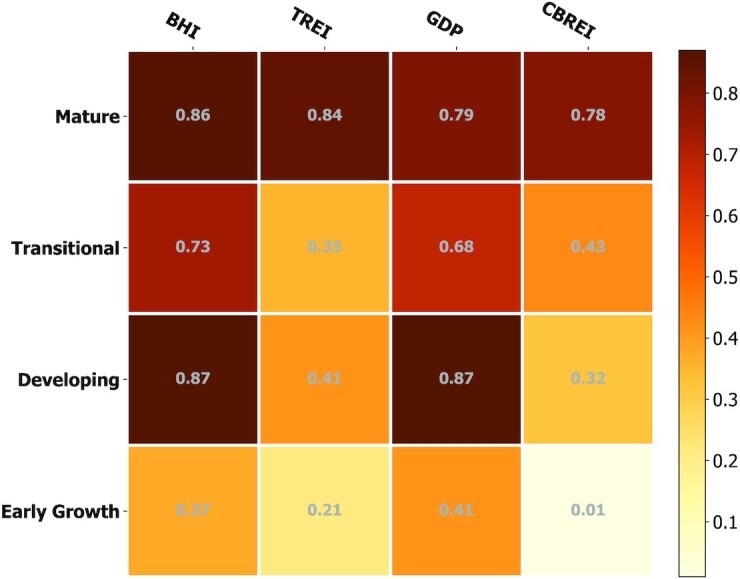
Correlation of PageRank with socioeconomic indicators BHI, TREI, GDP, and CBREI. Correlation strength is measured by Spearman’s coefficient ρ_*s*_.

Next, we disaggregate the cities based on their level of development and geographical region to check whether the results are consistent across these categories. In Fig. 2, we plot the PageRank against BHI for four levels of development: Mature, Transitional, Developing, and Early Growth. We find a progressive decrease in the strength of association from Mature cities (*ρ_s_* = 0.86) through Transitional and Developing cities (*ρ_s_* = 0.73 and 0.88, respectively) and finally a weak correlation in Early Growth cities (*ρ_s_* = 0.38). Indeed, the differences become rather stark when looking at the other socioeconomic indicators as seen in Fig. 3. In Mature cities, the PageRank is strongly correlated with all indicators, in Transitional cities the correlation with real-estate investment weakens significantly, and moving to the other two categories the correlation is weak with all indicators (see Fig. S6 for correlation summary of socioeconomic indicators with other network centrality measures).

Differences also exist when grouping cities based on their geographical location as shown in Fig. S5. In prosperous regions (North America, Australasia, and Western Europe) the association between PageRank and BHI is quite strong (ρ_*s*_ = 0.86, 0.93, and 0.76, respectively), in areas with mixed levels of prosperity such as Latin America, Asia, and the Middle East, the correlations are slightly weaker (ρ_*s*_ = 0.72, 0.86, and 0.73, respectively) with the weakest correlations being in Sub-Saharan Africa (ρ_*s*_ = 0.38).

We note, the possible confounding factor of agglomeration effects. Indeed, urban agglomeration is a major facilitator of socioeconomic development (69) by providing shareable inputs for businesses such as a common labor pool, technical expertise, general knowledge, personal contacts, and public infrastructure (70). On the other hand, intercity connections and long-distance trips are crucial driving forces of urban economic welfare by promoting international economic activity, as movement of people fosters the movement of capital (41). It is the latter that is captured by the mobility network and the associated PageRank centrality, and therefore does not include intracity agglomeration effects. Indeed, a key metric of agglomeration is population size; a multivariate analysis shown in Table S2 indicates that population does not play a significant role as a confounding variable influencing the association between PageRank and the socioeconomic indicators.

### Connection of core–periphery organization to urban welfare

To check whether these observed differences based on maturity and location are consequences of higher-order topological features of the mobility network, we next employ the *k-core* decomposition, that identifies strongly connected core-nodes and sparsely connected peripheral nodes, revealing meso-scale network structure (71,72). Applying this analysis, we find two core clusters consisting of 49 Western European cities and 40 North American cities as shown in Fig. S7. The rest of the clusters are much smaller (sizes between 5 and 20) and lie in the peripheral layers of the mobility network. The role of a city’s position in the inner or outer-layers becomes clear when plotting ρ_*s*_ between PageRank and BHI as a function of the layer-number *k* (numbered in increasing order from the outer to inner layer) as shown in Fig. S8. We see a monotonically decreasing trend of the strength of correlation as one moves from the core to the peripheral layers, indicating that PageRank become a poor predictor of welfare for cities located more in the outer-layers of the mobility network.

The existence of large core clusters concentrated in Western Europe and North America (all Mature or Transitional cities) suggest that they form a densely interconnected backbone of the mobility network and thus control most of human capital and resource flow. This can be quantified by studying the weighted rich-club organization (73), which is defined as

**Fig. 4.**
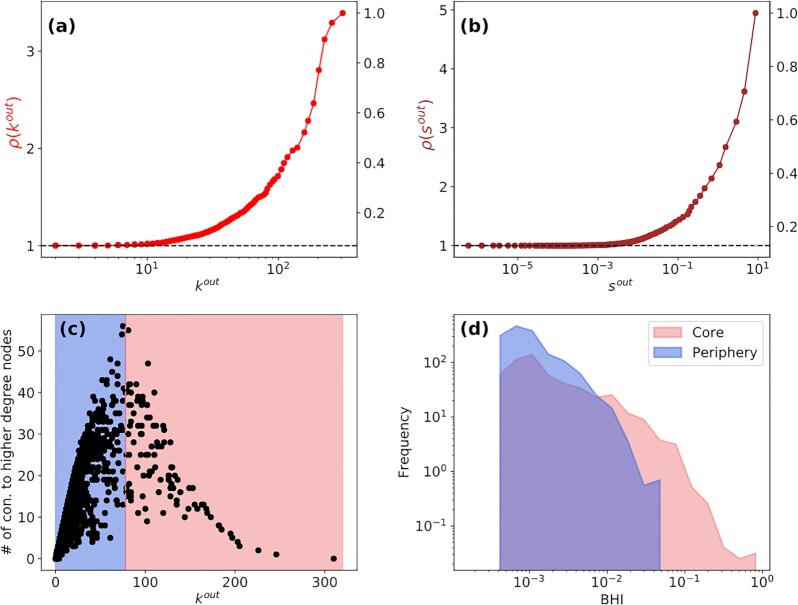
Rich-club organization and core–periphery structure of the mobility network. The richness parameter ρ(*r*) (Eq. 3) (a) for the connectivity *r* = *k*^*out*^ and (b) weighted out-degree *r* = *s*^out^. The monotonically increasing trend of ρ(*r*) with *r* indicates subsets of high-connectivity cities that connect to each other more than would be expected as merely a consequence of the distribution of links and edge-weights. (c) The number of neighbors of each node, with a higher out-degree than the node itself, as a function of the node’s out-degree. The scatter plot has a peak separating cities into two clusters of core (in red) and peripheral (in blue) nodes. (d) The distribution of BHI in the core and peripheral cities, showing that cities in the core typically have much higher values of BHI than those in the periphery.

(3) \begin{equation*} \rho (r)=\frac{\phi (r)}{\phi _{\text{null }}(r)}, \end{equation*}

where *r* is a *richness parameter*. Two flavors of the parameter that we consider are the out-degree *k*^out^ and the out-strength *s*^out^. Given this, ϕ(*k*) is a fraction that measures the number of edges between nodes of degree ≥*k*^out^ compared to the edges they would share if all these nodes were connected to each other. The quantity ϕ_null_(*k*^out^) is the corresponding measure under out degree-preserving reshuffling of links. For *s*^out^, ϕ_null_(*s*^out^) is computed through both edge and weight reshuffling. Values of ρ(*r*) > 1 indicate a set of nodes that are connected to more cities, with higher exchange of populations, than one would expect merely as a consequence of the distribution of links and edge-weights. In Fig. 4(a), we plot ρ(*k*^out^) and in Fig. 4(b)*ρ*(*s*^out^), finding that nodes with high connectivity and strength of connections are between two to three times more connected to each other than would be expected by random chance. The results indicate that Mature and Transitional cities form a *rich-club* dominating mobility flows in the network.

Finally, while the *k*-core decomposition allows for assigning nodes to layers, there is no clear way to define a boundary between the core and periphery. To do so, we use a method introduced in (74): first, we sort nodes with respect to *k*^out^ in increasing order. Then, for each node, we count the number of its neighbors that have out-degree higher than itself. In Fig. 4(c), we plot the sorted degrees of all nodes and their corresponding number of connections to higher-degree nodes. There is a clear peak in the scatter plot that allows us to distinguish two clusters of cities being in the core (in red) and in the periphery (in blue) (75). The figure indicates that the top $~30\%$ of nodes in terms of their out-degree are in the core of the mobility network, consistent with the two dense cores identified by the *k* −core method in Fig. S8. Having identified the core and the periphery, we plot the distribution of BHI in the two regions of the network in Fig. 4(d) finding that cities in the core are in general more prosperous than peripheral cities.

### Relationship between welfare and mobility neighborhood

The results, thus far point towards strongly interconnected sets of core cities that dominate mobility flow (and therefore exchange of human resources) that are successful in terms of socioeconomic indicators. This success can be reasonably well predicted by their centrality, or just their topological features in relation to the mobility network. For cities in the periphery, and those that are not so tightly connected, their level of development cannot be simply captured by topological aspects. In particular PageRank is a poor measure to capture such cities’ welfare, given that it is noisy for nodes with low connectivity and edge-weights (61).

Furthermore, it does not capture an important feature, that is the maturity level of the cities from which travelers come to a given city. For instance, it stands to reason that for two Developing cities with similar connectivity profiles, if one of them has visitors from Mature cities, and the other only from Developing cities, then the former may benefit more from the flow of intellectual and human capital from more developed regions. To account for this effect, we define the quantity

(4) \begin{equation*} \langle S\rangle _i=\frac{\sum \limits _j W^I_{ji} S_j}{\sum \limits _j W^I_{ji}}\ , \end{equation*}

where *S_j_* denotes the success/welfare of a given city *j* (measured through the socioeconomic indicators) and W^I^ contains only international flows from *j* to *i*. The quantity 〈*S*〉_*i*_ can then be interpreted as the weighted average of the success of the international visitors to city *i*. For instance, if we take BHI as the measure of success, then this number will be high if most visitors are from Mature cities, and low if they are from Early Growth cities. Based on this, we define an estimator for a city *i*’s success.

(5) \begin{equation*} \hat{S}_i = PR_i \times \langle S\rangle _i^\gamma , \end{equation*}

where *PR_i_* is its PageRank and *γ* is a tunable parameter that measures the extent to which the level of development of a city’s neighbors plays any role in predicting its own development. For *γ* ≃ 0, the node’s network topological properties are the best indicator of its welfare, whereas for *γ* ≥ 0 incorporating information about the development levels of its neighbors provides a better estimate.

Table 1 contains the optimal value of the exponent *γ* (see Figs. S9 and S10), maximizing Spearman’s correlation ρ_*s*_(*γ*), between the estimator $\hat{S}_i$, and a city’s actual level of success *S_i_* as quantified by the BHI which captures the city’s maturity level. We note that the analysis excludes six cities in the USA (Birmingham, Kansas, Omaha, Stamford, and Richmond) and two from Mexico (Puebla and Querétaro). For these cities there is no BHI data on any of their international connections. The role of the average success of neighbors 〈 *S*〉 is the most prominent for Early Growth cities, and practically non-existent for Mature cities (see Fig. S11 for in-flow breakdown of Early Growth and Developing cities originating from national and international cities). Table   2 contains the same information for cities categorized by geographical region. Mature cities in Western Europe and North America display the lowest values for *γ*, whereas regions such as Asia, Latin America, and the Caribbean—that contain a mix of cities in terms of their development levels—show intermediate values for *γ*. The highest values are seen for the Middle East region and Central Europe that are home to fast rising cities. Interestingly, Sub-Saharan Africa shows the same trend as Asia, though it is an outlier, as it seems neither the PageRank nor the development levels of its neighbors are enough to account for explaining its own levels of development (ρ_*s*_(*γ*) = 0.47) unlike for the other regions (ρ_*s*_(*γ*) ≥ 0.76).

**Table 1.** Role of mobility neighbourhood for cities group by their maturity. γ^*opt*^ corresponds to the γ value optimizing Spearman’s correlation coefficient between the actual success *S_i_* (BHI) and the estimator $\hat{S}_i$ Eq. (5). Size denotes the number of cities in each category. ρ_*s*_(γ) corresponds to Spearman’s coefficient given a γ value. The brackets show the 95% CI computed by performing an empirical bootstrapping of the data (76). For each group, the CIs are computed by sampling 50 data subsets comprising $85\%$ of its cities.

Development-stage	Size	γ^*opt*^	ρ_*s*_(γ^*opt*^)	ρ_*s*_(0)
Early Growth	64	0.35 (−0.40 to 1.81)	0.22 (0.17 to 0.31)	0.21 (0.14 to 0.29)
Mature	131	0.07 (−0.00 to 0.29)	0.65 (0.62 to 0.69)	0.65 (0.61 to 0.68)
Transitional	29	−0.00 (−0.49 to 0.03)	0.61 (0.50 to 0.75)	0.61 (0.50 to 0.73)
Development	44	−0.07 (−0.67 to 0.05)	0.78 (0.75 to 0.84)	0.78 (0.74 to 0.82)

**Table 2.** Role of mobility neighborhood for cities grouped by geographic region.

Region	Size	γ^*opt*^	ρ_*s*_(γ^*opt*^)	ρ_*s*_(0)
CEE/CIS	27	2.48 (1.64 to 3.01)	0.70 (0.62 to 0.78)	0.56 (0.46 to 0.70)
LATAM/Caribbean	30	0.32 (0.18 to 0.93)	0.47 (0.30 to 0.64)	0.41 (0.24 to 0.57)
Western Europe	69	0.27 (0.15 to 0.38)	0.59 (0.52 to 0.66)	0.55 (0.48 to 0.64)
Asia	36	−0.05 (−0.19 to 0.20)	0.80 (0.76 to 0.86)	0.79 (0.75 to 0.84)
North America	64	−0.05 (−0.60 to 0.01)	0.64 (0.57 to 0.71)	0.64 (0.57 to 0.71)
MENA	18	−0.73 (−0.99 to 0.30)	0.57 (0.47 to 0.77)	0.51 (0.36 to 0.69)
Australasia	8	−1.20 (−3.00 to 4.10)	0.74 (0.66 to 0.94)	0.74 (0.54 to 0.83)
Sub-Saharan Africa	16	−3.00 (−3.00 to 5.19)	0.01 (−0.07 to 0.22)	−0.07 (−0.31 to 0.11)

All quantities, same as in Table 1.

### Role of success on mobility flows

The enhanced ability to predict the true socioeconomic indicator of a city in the less developed categories by incorporating the maturity level of its neighborhood suggests that perhaps success plays a role in how flows are allocated. To check for this, we construct a simple model for the nature of outgoing flows of a city among its destinations that accounts for both the success of the target destinations, as well as the distance between these targets and the source city. Suppose a city *i* has a certain amount of total outflow *c_i_* which is incident on different cities *j* within some set of target cities ∂_*i*_—its mobility network neighborhood. We assume that city *i*’s outgoing flow *c_i_* to neighbors *j* ∈ ∂_*i*_ is partitioned based on the success *S_j_* of city *j*, as well as the distance *d_ij_*, so that $\tilde{T}_{ij}(\mu ,\nu )\propto S_j^{\mu }d_{ij}^{\nu }$, where *μ* and *ν* are free parameters, and $\tilde{T}_{ij}(\mu ,\nu )$ is the predicted flow from *i* to *j* from the model (whose dependence on *μ* and *ν* has been made explicit). More precisely, we have

(6) $$\begin{eqnarray*}
\tilde{T}_{ij}(\mu ,\nu ) = \frac{S_j^{\mu }d_{ij}^{\nu }}{\sum _{j\in \partial _i}S_j^{\mu }d_{ij}^{\nu }}\times c_i.
\end{eqnarray*}
$$

If the success *S_j_* makes travel to city *j* more attractive to travelers from city *i*, then one would expect *μ* ≥ 0. On the other hand, if the cities are far apart in distance *d_ij_*, then it would make it more costly to travel from *i* to *j*, and in that case *ν* ≤ 0. The ratio $|\frac{\mu }{\nu }|$ then determines the relative importance of these factors in determining $\tilde{T}_{ij}$. Given that $\tilde{T}_{ij}(\mu ,\nu )$ is invariant to the scale of *S_j_* and *d_ij_*, the relative importance of these measures can be compared directly using this ratio regardless of units.

The outflow *c_i_* can be inferred from the raw (unweighted) origin–destination matrix $\boldsymbol {T}$ using the expression $c_i=\sum _{j\in \partial _i}T_{ij}$, where the neighborhood ∂_*i*_ is restricted to the subset of cities *j* that *i* connects to that also have BHI metadata *S_j_*. We then fit Eq. (6) to the true observed flows *T_ij_* to find the exponents $\hat{\mu }$ and $\hat{\nu }$ that optimize the Pearson correlation between the true and predicted flows *T_ij_* and $\tilde{T}_{ij}$ for connected city pairs *i* and *j*. This optimization is nontrivial but can be done approximately with a variety of methods, and here we choose a basin hopping algorithm (77). Higher values of $|\hat{\mu }/\hat{\nu }|$ indicate that outflows are strongly associated with success, and low values of $|\hat{\mu }/\hat{\nu }|$ indicate that outflows are much more strongly associated with distance than with success. To determine whether the city subgroup (development stage or geographical region) affects the association between success and outgoing flows, we identify separate exponents $\lbrace \hat{\mu },\hat{\nu }\rbrace$ for each subgroup by optimizing the Pearson correlation between $\tilde{T}_{ij}$ and *T_ij_* for all flows leaving cities *i* in the subgroup.

We also determine whether or not the success covariate *S_j_* (BHI) significantly improves our predictive model of outflows over a baseline model, where only the distance between cities is considered. To do this, we identify the exponent *ν*_0_ that optimizes the correlation between the true and predicted flows while ignoring the success parameter (*μ* = 0). Since the model with *μ* has one more free parameter than the model with *μ* = 0, the fit between the true and predicted flows will always be better according to the Pearson correlation ρ_*p*_. Thus for a fair comparison, the more complex model needs to be penalized using some model selection criteria. Here we opt for both the Akaike Information Criterion (AIC) and the Bayesian Information Criterion (BIC) (78), which are derived from the log-likelihood of the model fit, as well as the number of data points in the sample. Table S3 includes all model fit results for each category of cities. Applying both penalties indicate that the model performs significantly better with the inclusion of *μ* for all subgroups. We find that $\hat{\mu }\gt 0$ and $\hat{\nu }\lt 0$ for all city subgroups, indicating that outflows are positively associated with higher values of BHI and negatively associated with distance, though the extent to which this happens depends on the level of development, with the effect most pronounced in Early Growth and Developing cities, and least so in Mature and Transitional cities.

**Fig. 5.**
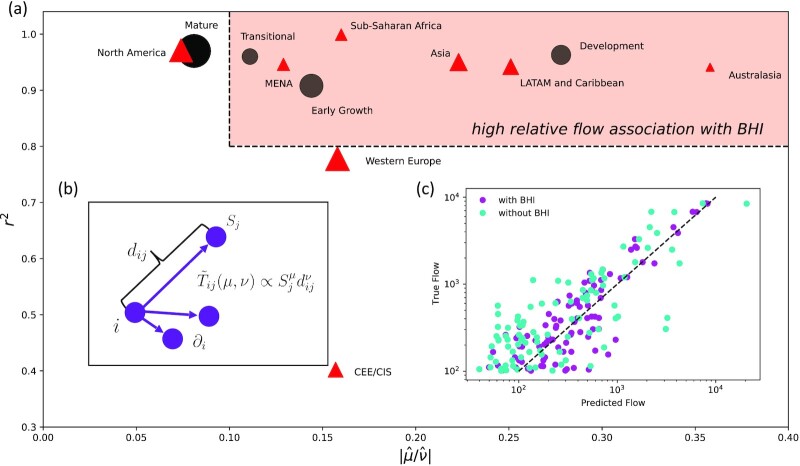
Interplay between benefit (success) and cost (distance). (a) $|\hat{\mu }/\hat{\nu }|$ and coefficient of determination *r*^2^ for all city subgroups considered, using the model in Eq. (6) to fit the outflows of all cities in the subgroup. Black circles indicate development subgroups, and red triangles indicate geographical subgroups, while the size of the marker is proportional to the number of cities in the subgroup. Cities with *r*^2^ > 0.8 and $|\hat{\mu }/\hat{\nu }|\gt 0.1$ are highlighted as having high relative flow association with BHI due to high model correlations and contributions from $S_j^\mu$. All fits demonstrated significant improvement through the inclusion of the $S_j^\mu$ term, as evidenced by the corresponding AIC and BIC values (Table S3 in Supplementary Material). (b) Model schematic, showing the variables involved in Eq. (6) around a central node *i* for which outflows $\tilde{T}_{ij}$ are being predicted. (c) Example predicted and true outflows from cities in the Australasia subgroup, showing significant improvement from including the BHI in the model fit. Only flows with *T_ij_* > 100 are displayed, for clearer visualization, and the line of equality is shown for reference.

In Fig. 5(a), the relative magnitude $|\hat{\mu }/\hat{\nu }|$ of the coefficients $\hat{\mu }$ and $\hat{\nu }$ is plotted along with the coefficient of determination *r*^2^ for each city subcategory. As discussed, the ratio $|\hat{\mu }/\hat{\nu }|$ quantifies the relative magnitude of association of success *S_j_* and distance *d_ij_* with the outflows *c_i_* from city *i*. According to the model fits, in general outflows from Developing cities are more strongly associated with success than outflows from more developed cities, as indicated by the shaded red box. More specifically, outflows from cities in the Developing, Early Growth, and Transitional subgroup have a higher relative magnitude of association with the success of the cities they flow to, as compared to outflows from cities in the Mature subgroup. We also see a geographic dependence with outflows from cities in North America and (Western and Eastern) Europe having lower relative levels of association with BHI and having weaker model fits in general. This suggests that other factors are more important in determining the corresponding flows (perhaps consistent with these regions having many developed cities). As insets, we illustrate the variables used in the flow model (Fig. 5b), as well as show an example of the improved weight prediction after the inclusion of BHI for the Australasia subgroup (Fig. 5c). In this third panel, we plot the flow as predicted by Eq. (6)—with $\hat{\mu }$ and $\hat{\nu }$ as the model parameters—along the *x*-axis, and the true observed flow along the *y*-axis. The weights condense significantly around the line of equality when including *S_j_* as a co-variate, as compared to using only *d_ij_*. These general patterns are robust to the success measure *S_j_* used, as can be seen in Table S4, where we have repeated the analysis with *S_j_* set to GDP instead of BHI.

These results indicate that outflows from cities in earlier stages of development or in more developing regions are more highly associated with the success of cities they are incident upon. These results complement our previous analysis by suggesting that perhaps individuals in such cities strategically chose locations to visit in order to maximize the benefits they receive by connecting with more successful cities. On the other hand, for developed cities outflows are more strongly associated with travel distance.

## Discussion

We have presented here, a comprehensive analysis on the connection between intercity global mobility flows and the welfare and success of urban areas. At the macroscopic level, we report the existence of a tightly connected core of cities that form a rich-club dominating mobility flow in the network. Cities in this core tend to have higher levels of development, and are primarily located in Western Europe and North America. For these cities, their development-level is well predicted by their network topological properties, in particular centrality measures such as PageRank. On the other hand, Developing and Early Growth cities are located in the peripheral layers and are scattered across multiple regions (Asia, Eastern Europe, Latin America, Middle East, and Sub-Saharan Africa). Their level of development is partially connected to their network centrality in some cases, and very weakly so in others, in particular in Africa.

For those regions, whose success is weakly connected to their centrality, we find that adding information on their mobility neighborhood (i.e. the average development level of the cities they are connected to) leads to a marked increase in correlations with their socioeconomic indicators. This effect is less pronounced for more developed cities, whose socioeconomic indicators are connected primarily with their network centrality. Once again Sub-Saharan Africa is an outlier, its development being weakly connected to network properties and the socioeconomics of their mobility neighborhood. To check whether the connection of cities to other cities is indeed influenced by their development-level, we propose a simple model that disentangles the relevant aspects influencing the success/welfare of urban areas. The model assumes that the outflow of cities is distributed among destinations in proportion to their relative benefit (maturity) and cost (distance). The predicted outflows are compared to the empirical outflows to determine the contribution of each individual component of the model. We find that all cities incorporate the development level of the cities they connect to, and are less inclined to connect to cities at great distance. However, the former is much more pronounced in developing regions as compared to developed regions.

Taken together, our analysis indicates that the welfare of cities and the interurban mobility network are strongly correlated, but that this correlation depends on their level of maturity. The global urban ecosystem appears to be a combination of a well-established set of core urban areas whose interconnections describe their performance according to network science principles, and a subset of Developing cities whose welfare is influenced by the extent of connections to the core. The core set of cities also exhibit a rich-club phenomenon, whereby the density of internal connections within the core is higher than expected, which might be related to the existence of global cities as proposed in sociological studies (79).

While we do not have data on individual mobility-flows the results are akin to that seen in (21), where artists climb the ladder, as it were, by strategically positioning themselves in the network of galleries exhibiting their work. Indeed, the observation that the success of Developing cities is better predicted by including information on their mobility neighborhood, suggests that such cities rise up the “value-chain” by leveraging their neighborhood for the exchange of human capital and resources. Mature cities on the other hand are already in an advanced stage of development and the effects of their mobility neighborhood are diminished in time. This, however, can only be checked with longitudinal data, the availability of which will allow for more detailed analysis. In conclusion, our findings suggest that intercity mobility must also be incorporated as one of the factors that influence urban growth.

## Limitations

These results should be interpreted in light of several important limitations. First, the Google mobility data are limited to smartphone users who have opted in to Google Location History feature, which is off by default. These data may not be representative of the population as whole, and may vary by location. Importantly, these limited data are only viewed through the lens of differential privacy algorithms, specifically designed to protect user anonymity and obscure fine detail. Moreover, comparisons across, rather than within locations are only descriptive since these regions can differ in substantial ways. For instance, privacy choices and income thresholds may vary from country to country. Nevertheless, it has been shown that the dataset provides equal coverage across populations irrespective of their income profiles (12).

Another limitation is the fact that the dataset does not provide information about the composition of population flows. Namely, the fraction of capital, knowledge, and trade flows cannot be distinguished in the mobility network, given that it is aggregated across all types of flow. This precludes one from making more granular analysis on the differences between trade and population flows, for instance. Furthermore, due to the static nature of the network, temporary and permanent migration patterns of human labor cannot be identified. This introduces an issue of endogeneity, whereby it is difficult to ascertain whether migration creates better economic conditions, or is it that migrants tend to move to locations that already are economically attractive. Likewise, our analysis overlooks other socioeconomic factors beyond mobility which might also play a crucial role to shape urban welfare. Availability of longitudinal data and the ability to distinguish between the types of flow will prove invaluable for more detailed investigations. Finally, the exclusion of China from the analysis will necessarily affect the set of cities identified to be part of the well-developed urban core.

## Supplementary Material

pgac178_Supplemental_File

## Data Availability

The Google Aggregated Mobility Research Dataset used for this study is available with permission from Google LLC. Enquiries should be sent to the corresponding author(s). Links to all other data sources have been provided in the manuscript."
