# Supplementary material for: Connecting intercity mobility with urban welfare
Source: PNAS Nexus. 2022 Sep 7;1(4):pgac178. doi: 10.1093/pnasnexus/pgac178 (PMC9802375; doi:10.1093/pnasnexus/pgac178)
Supplement: pgac178_Supplemental_File [file pgac178_supplemental_file.pdf]

Supporting Information

The impact of inter-city mobility on urban welfare

S. Mimar et al.

Table of Contents

S1 Global cities with socioeconomic indicators **2**

S2 Comparison of PageRank and strength correlations with BHI **10**

S3 Categorical comparison **11**

S4 Effect of Population **12**

S5 Core-Periphery Structure **13**

S6 Exponent γ vs correlation coefficients **15**

List of Figures

S1 Cities selected for the study . . . 2
S2 Histograms of cities in subcategories . . . 2
S3 Dependence of correlation coefficients on the weight exponent β . . . 10
S4 Global Correlation Summary . . . 10
S5 Pagerank vs BHI at geographic region level . . . 11
S6 Correlation Summary at Development Level . . . 12
S7 Core cities . . . 13
S8 Correlation on core-periphery structure . . . 14
S9 Dependence of the Spearman's and Pearson correlation coefficient on the exponent γ . 15
S10 Dependence of the Spearman's and Pearson's correlation coefficient ρ_P on the exponent γ 16
S11 In-flow distribution of Early Growth and Developing cities . . . 17

List of Tables

S1 List of cities with their Geographic Region and Development-Stage. . . . 3
S2 Effect of Population . . . 13
S3 Model Summary 1 . . . 17
S4 Model Summary 2 . . . 18

S1 Global cities with socioeconomic indicators

In this study, we use 268 urban areas that have socioeconomic indicators (BHI, GDP, Total Real Estate Investment,Total Cross boundary investment), published by JLL and illustrated in the map below S1 and listed in Table S1. In addition to the socioeconomic indicators, cities are categorized with respect to their development level and geographic location. In Fig. S2, we show number of urban areas in each subcategory.

The cities in the dataset house a combined population of 1.3 billion, account for nearly 40% of global economic activity, contain 88% of global headquarters for the 2,000 largest listed companies, generate 40% of the world's finance and business employment, and more than 80% of global air passenger traffic (`https://seoulsolution.kr/sites/default/files/gettoknowus/jll-global300-2015.pdf`).

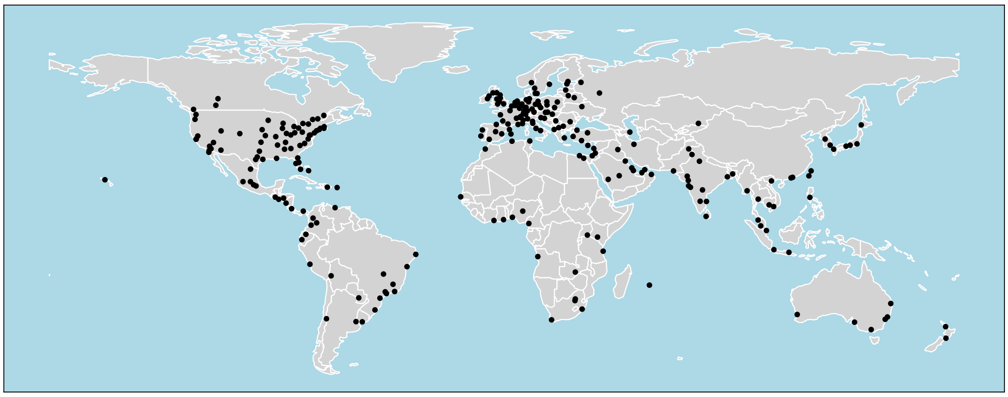

Figure S1: Cities selected for the study

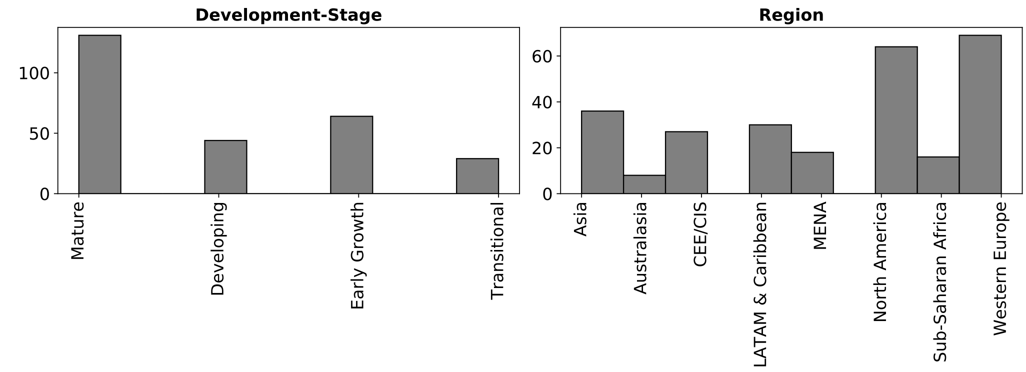

Figure S2: Histograms of cities in subcategories

Table S1: List of cities with their Geographic Region and Development-Stage.

City	Country	Region	Development-Stage
Surat	India	Asia	Early Growth
Tokyo	Japan	Asia	Mature
Lahore	Pakistan	Asia	Early Growth
Nagoya	Japan	Asia	Transitional
Hong Kong	Hong Kong	Asia	Mature
Pune	India	Asia	Developing
Singapore	Singapore	Asia	Mature
Osaka	Japan	Asia	Mature
Jakarta	Indonesia	Asia	Developing
Seoul	South Korea	Asia	Transitional
Ahmedabad	India	Asia	Early Growth
Dhaka	Bangladesh	Asia	Early Growth
Penang-Georgetown	Malaysia	Asia	Developing
Colombo	Sri Lanka	Asia	Early Growth
Yangon	Myanmar	Asia	Early Growth
Hanoi	Vietnam	Asia	Early Growth
Bangalore	India	Asia	Developing
Sapporo	Japan	Asia	Transitional
Chennai	India	Asia	Developing
Kaohsiung	Taiwan	Asia	Developing
Karachi	Pakistan	Asia	Early Growth
Ho Chi Minh City	Vietnam	Asia	Early Growth
Kolkata	India	Asia	Developing
Taipei	Taiwan	Asia	Transitional
Busan	South Korea	Asia	Developing
Surabaya	Indonesia	Asia	Early Growth
Fukuoka	Japan	Asia	Transitional
Manila	Philippines	Asia	Developing
Kuala Lumpur	Malaysia	Asia	Transitional
Delhi	India	Asia	Developing
Hyderabad	India	Asia	Developing
Bangkok	Thailand	Asia	Developing
Islamabad	Pakistan	Asia	Early Growth
Mumbai	India	Asia	Developing
Macau	Macau	Asia	Developing
Phnom Penh	Cambodia	Asia	Early Growth
Brisbane	Australia	Australasia	Mature

Wellington	New Zealand	Australasia	Mature
Perth	Australia	Australasia	Mature
Adelaide	Australia	Australasia	Mature
Newcastle	Australia	Australasia	Mature
Sydney	Australia	Australasia	Mature
Auckland	New Zealand	Australasia	Mature
Melbourne	Australia	Australasia	Mature
Tirana	Albania	CEE/CIS	Early Growth
Istanbul	Turkey	CEE/CIS	Developing
Skopje	Macedonia	CEE/CIS	Early Growth
Minsk	Belarus	CEE/CIS	Early Growth
Poznan	Poland	CEE/CIS	Developing
Antalya	Turkey	CEE/CIS	Early Growth
Moscow	Russia	CEE/CIS	Transitional
Almaty	Kazakhstan	CEE/CIS	Early Growth
Izmir	Turkey	CEE/CIS	Early Growth
Ankara	Turkey	CEE/CIS	Developing
Baku	Azerbaijan	CEE/CIS	Early Growth
Prague	Czech Republic	CEE/CIS	Transitional
Kiev	Ukraine	CEE/CIS	Developing
Vilnius	Lithuania	CEE/CIS	Transitional
Sofia	Bulgaria	CEE/CIS	Developing
Riga	Latvia	CEE/CIS	Transitional
Krakow	Poland	CEE/CIS	Developing
Zagreb	Croatia	CEE/CIS	Early Growth
Tallinn	Estonia	CEE/CIS	Transitional
Bucharest	Romania	CEE/CIS	Developing
St Petersburg	Russia	CEE/CIS	Developing
Wroclaw	Poland	CEE/CIS	Developing
Warsaw	Poland	CEE/CIS	Transitional
Budapest	Hungary	CEE/CIS	Transitional
Bratislava	Slovakia	CEE/CIS	Transitional
Ljubljana	Slovenia	CEE/CIS	Developing
Belgrade	Serbia	CEE/CIS	Early Growth
Porto Alegre	Brazil	LATAM/Caribbean	Early Growth
La Paz	Bolivia	LATAM/Caribbean	Early Growth
Asuncion	Paraguay	LATAM/Caribbean	Early Growth
Campinas	Brazil	LATAM/Caribbean	Early Growth
Rio de Janeiro	Brazil	LATAM/Caribbean	Developing
Bogota	Colombia	LATAM/Caribbean	Developing
Santiago	Chile	LATAM/Caribbean	Transitional

San Jose	Costa Rica	LATAM/Caribbean	Developing
Medellin	Colombia	LATAM/Caribbean	Early Growth
Curitiba	Brazil	LATAM/Caribbean	Early Growth
Belo Horizonte	Brazil	LATAM/Caribbean	Developing
Sao Paulo	Brazil	LATAM/Caribbean	Developing
Cali	Colombia	LATAM/Caribbean	Early Growth
Panama City	Panama	LATAM/Caribbean	Early Growth
Quito	Ecuador	LATAM/Caribbean	Early Growth
Nassau	Bahamas	LATAM/Caribbean	Transitional
Guatemala City	Guatemala	LATAM/Caribbean	Early Growth
Montevideo	Uruguay	LATAM/Caribbean	Developing
Tegucigalpa	Honduras	LATAM/Caribbean	Early Growth
Caracas	Venezuela	LATAM/Caribbean	Early Growth
Buenos Aires	Argentina	LATAM/Caribbean	Developing
Santo Domingo	Dominican Republic	LATAM/Caribbean	Early Growth
Guayaquil	Ecuador	LATAM/Caribbean	Early Growth
Recife	Brazil	LATAM/Caribbean	Early Growth
Managua	Nicaragua	LATAM/Caribbean	Early Growth
San Juan	Puerto Rico	LATAM/Caribbean	Developing
Salvador	Brazil	LATAM/Caribbean	Early Growth
San Salvador	El Salvador	LATAM/Caribbean	Early Growth
Brasilia	Brazil	LATAM/Caribbean	Developing
Lima	Peru	LATAM/Caribbean	Early Growth
Dubai	United Arab Emirates	MENA	Developing
Baghdad	Iraq	MENA	Early Growth
Jeddah	Saudi Arabia	MENA	Early Growth
Doha	Qatar	MENA	Developing
Amman	Jordan	MENA	Early Growth
Abu Dhabi	United Arab Emirates	MENA	Developing
Algiers	Algeria	MENA	Early Growth
Cairo	Egypt	MENA	Early Growth
Alexandria	Egypt	MENA	Early Growth
Kuwait City	Kuwait	MENA	Early Growth
Tunis	Tunisia	MENA	Early Growth
Tehran	Iran	MENA	Early Growth
Casablanca	Morocco	MENA	Developing
Manama	Bahrain	MENA	Developing
Riyadh	Saudi Arabia	MENA	Early Growth
Tel Aviv	Israel	MENA	Transitional
Beirut	Lebanon	MENA	Early Growth
Muscat	Oman	MENA	Early Growth

Columbus	USA	North America	Mature
Las Vegas	USA	North America	Mature
Austin	USA	North America	Mature
Raleigh-Durham	USA	North America	Mature
Kansas City	USA	North America	Mature
Milwaukee	USA	North America	Mature
Salt Lake City	USA	North America	Mature
New York	USA	North America	Mature
Pittsburgh	USA	North America	Mature
Memphis	USA	North America	Mature
Quebec City	Canada	North America	Mature
Puebla	Mexico	North America	Early Growth
Stamford- Bridgeport-Norwalk	USA	North America	Mature
Rochester	USA	North America	Mature
Birmingham	USA	North America	Mature
Tulsa	USA	North America	Mature
Queretaro	Mexico	North America	Developing
Omaha	USA	North America	Mature
Providence	USA	North America	Mature
Jacksonville	USA	North America	Mature
Tijuana	Mexico	North America	Developing
Indianapolis	USA	North America	Mature
Riverside-San Bernardino	USA	North America	Mature
New Orleans	USA	North America	Mature
Richmond	USA	North America	Mature
Edmonton	Canada	North America	Mature
Ottawa	Canada	North America	Mature
Sacramento	USA	North America	Mature
San Antonio	USA	North America	Mature
Nashville	USA	North America	Mature
Honolulu	USA	North America	Mature
Hartford	USA	North America	Mature
Cincinnati	USA	North America	Mature
Guadalajara	Mexico	North America	Developing
Baltimore	USA	North America	Mature
Boston	USA	North America	Mature
Cleveland	USA	North America	Mature
Tampa	USA	North America	Mature
St Louis	USA	North America	Mature
Dallas	USA	North America	Mature
Monterrey	Mexico	North America	Developing

San Francisco	USA	North America	Mature
Atlanta	USA	North America	Mature
Vancouver	Canada	North America	Mature
Washington	USA	North America	Mature
Calgary	Canada	North America	Mature
Mexico City	Mexico	North America	Developing
Seattle	USA	North America	Mature
Charlotte	USA	North America	Mature
Toronto	Canada	North America	Mature
Miami	USA	North America	Mature
Houston	USA	North America	Mature
San Jose	USA	North America	Mature
Philadelphia	USA	North America	Mature
Detroit	USA	North America	Mature
Orlando	USA	North America	Mature
San Diego	USA	North America	Mature
Phoenix	USA	North America	Mature
Portland	USA	North America	Mature
Los Angeles	USA	North America	Mature
Denver	USA	North America	Mature
Montreal	Canada	North America	Mature
Chicago	USA	North America	Mature
Minneapolis	USA	North America	Mature
Cape Town	South Africa	Sub-Saharan Africa	Transitional
Pretoria	South Africa	Sub-Saharan Africa	Transitional
Durban	South Africa	Sub-Saharan Africa	Transitional
Lagos	Nigeria	Sub-Saharan Africa	Early Growth
Dar es Salaam	Tanzania	Sub-Saharan Africa	Early Growth
Dakar	Senegal	Sub-Saharan Africa	Early Growth
Accra	Ghana	Sub-Saharan Africa	Early Growth
Port Louis	Mauritius	Sub-Saharan Africa	Developing
Abidjan	Cote D'Ivoire	Sub-Saharan Africa	Early Growth
Kampala	Uganda	Sub-Saharan Africa	Early Growth
Lusaka	Zambia	Sub-Saharan Africa	Early Growth
Nairobi	Kenya	Sub-Saharan Africa	Early Growth
Luanda	Angola	Sub-Saharan Africa	Early Growth
Douala	Cameroon	Sub-Saharan Africa	Early Growth
Johannesburg	South Africa	Sub-Saharan Africa	Transitional
Abuja	Nigeria	Sub-Saharan Africa	Early Growth
Nuremburg	Germany	Western Europe	Mature
Malmo	Sweden	Western Europe	Mature

Florence	Italy	Western Europe	Mature
Lille	France	Western Europe	Mature
Dresden	Germany	Western Europe	Mature
Copenhagen	Denmark	Western Europe	Mature
Edinburgh	United Kingdom	Western Europe	Mature
Utrecht	Netherlands	Western Europe	Mature
Zurich	Switzerland	Western Europe	Mature
Lyon	France	Western Europe	Mature
Hamburg	Germany	Western Europe	Mature
Lausanne	Switzerland	Western Europe	Mature
Brussels	Belgium	Western Europe	Mature
Rome	Italy	Western Europe	Mature
Hannover	Germany	Western Europe	Mature
Turin	Italy	Western Europe	Mature
Vienna	Austria	Western Europe	Mature
Dublin	Ireland	Western Europe	Mature
Naples	Italy	Western Europe	Transitional
Lisbon	Portugal	Western Europe	Mature
Dusseldorf	Germany	Western Europe	Mature
Newcastle-upon-Tyne	United Kingdom	Western Europe	Mature
Oslo	Norway	Western Europe	Mature
Geneva	Switzerland	Western Europe	Mature
Rotterdam	Netherlands	Western Europe	Mature
Belfast	United Kingdom	Western Europe	Mature
Marseilles	France	Western Europe	Mature
Luxembourg	Luxembourg	Western Europe	Mature
Manchester	United Kingdom	Western Europe	Mature
Seville	Spain	Western Europe	Transitional
Stuttgart	Germany	Western Europe	Mature
Leipzig	Germany	Western Europe	Mature
Porto	Portugal	Western Europe	Transitional
Gothenburg	Sweden	Western Europe	Mature
Leeds	United Kingdom	Western Europe	Mature
Nantes	France	Western Europe	Mature
Birmingham	United Kingdom	Western Europe	Mature
Valencia	Spain	Western Europe	Transitional
Athens	Greece	Western Europe	Transitional
Helsinki	Finland	Western Europe	Mature
Bilbao	Spain	Western Europe	Transitional
Toulouse	France	Western Europe	Mature
Essen	Germany	Western Europe	Mature

Basel	Switzerland	Western Europe	Mature
Liverpool	United Kingdom	Western Europe	Mature
Bonn	Germany	Western Europe	Mature
Bremen	Germany	Western Europe	Mature
Madrid	Spain	Western Europe	Mature
Glasgow	United Kingdom	Western Europe	Mature
Milan	Italy	Western Europe	Mature
Nicosia	Cyprus	Western Europe	Transitional
Mannheim	Germany	Western Europe	Mature
Paris	France	Western Europe	Mature
Antwerp	Belgium	Western Europe	Mature
Bordeaux	France	Western Europe	Mature
Cologne	Germany	Western Europe	Mature
Bologna	Italy	Western Europe	Mature
Palma de Mallorca	Spain	Western Europe	Transitional
The Hague	Netherlands	Western Europe	Mature
Stockholm	Sweden	Western Europe	Mature
Bristol	United Kingdom	Western Europe	Mature
Berlin	Germany	Western Europe	Mature
London	United Kingdom	Western Europe	Mature
Amsterdam	Netherlands	Western Europe	Mature
Munich	Germany	Western Europe	Mature
Strasbourg	France	Western Europe	Mature
Nice	France	Western Europe	Mature
Frankfurt	Germany	Western Europe	Mature
Barcelona	Spain	Western Europe	Mature

S2 Comparison of PageRank and strength correlations with BHI

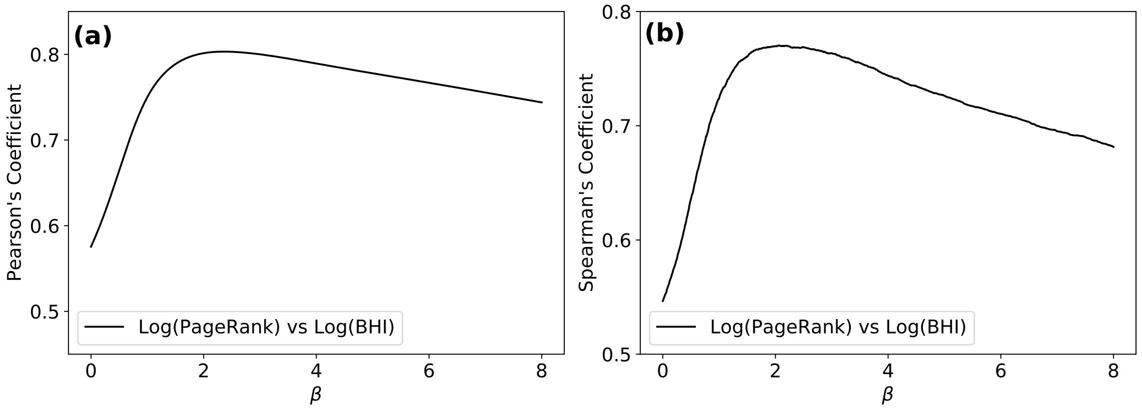

Figure S3: Pearson's in **a** and Spearman's in **b** correlation coefficient for Pagerank vs BHI as the distance exponent β is varied when computing the weights $W_{ij} = T_{ij} d_{ij}^{\beta}$. The curves yield a peak at $\beta = 2.5$ where the correlation coefficients are maximized.

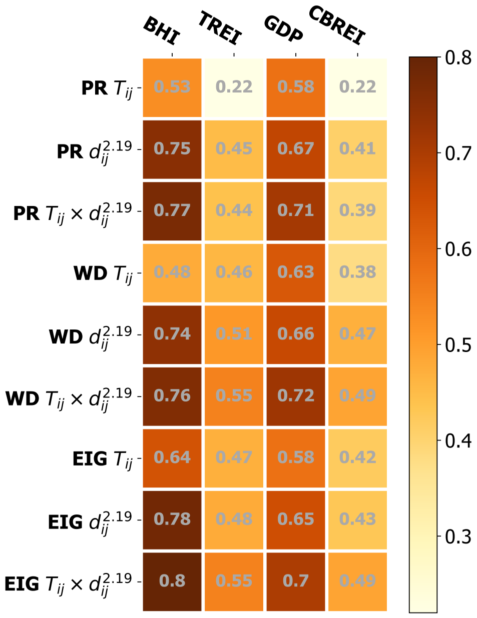

Figure S4: Correlation summary of mobility network centrality measures for global cities in the dataset, namely Pagerank (PR), Weighted-Degree (WD) and eigenvector centrality (EIG) versus socioeconomic indicators: BHI, Total real Estate investment, GDP and Cross-Border Real Estate Investment. Metrics are computed with various edge-weights to show the performance of flow (T_{ij}), distance d_{ij} and flow $\times$ distance ($T_{ij} d_{ij}$). The correlation metric used here is the Spearman's coefficient ρ_s.

S3 Categorical comparison

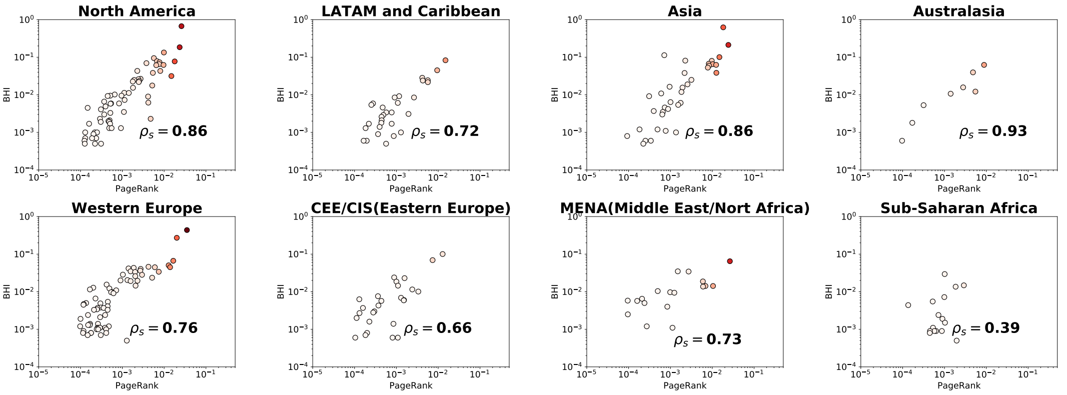

Figure S5: Pagerank vs BHI when cities are grouped according to geographic regions. Spearman's correlation in each panel shows association level of the socioeconomic indicator and the network centrality measure.Colors represent the strength of each city with dark red indicating high Weighted-Degree(WD).

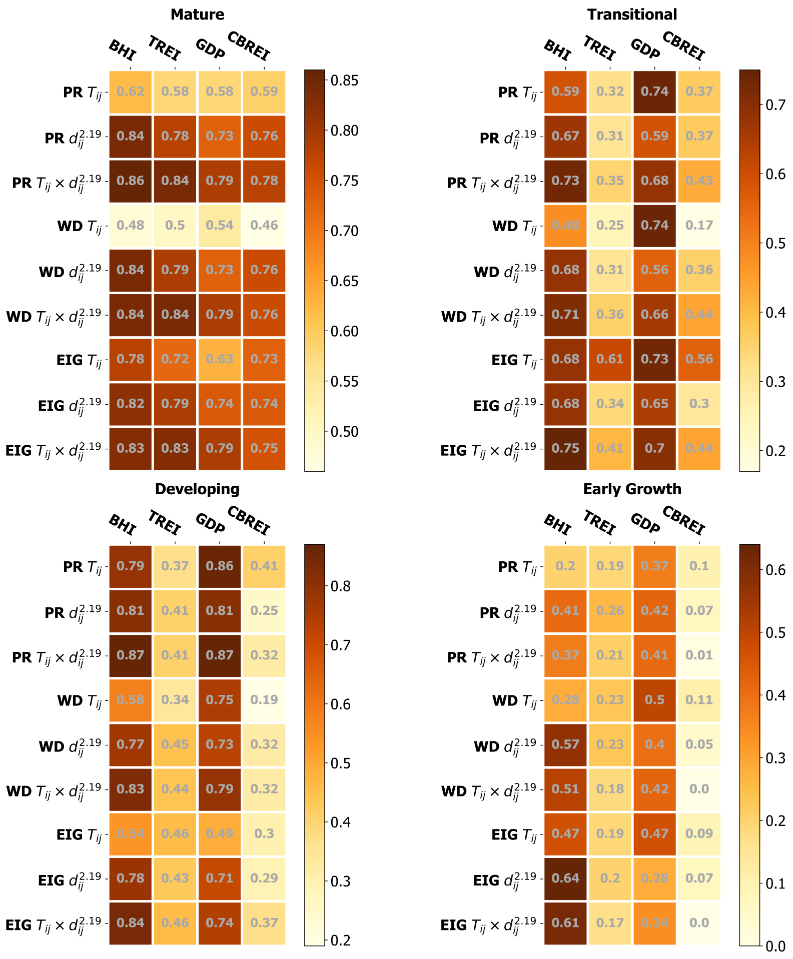

Figure S6: Correlation summary of mobility network centrality measures, namely Pagerank (PR), Weighted-Degree (WD) and eigenvector centrality (EIG) versus socioeconomic indicators: BHI, Total real Estate investment, GDP and Corss-Border Real Estate Investment. Metrics are computed with various edge-weights to show the performance of flow (T_{ij}), distance d_{ij} and flow × distance ($T_{ij}d_{ij}$). The correlation metric used here is the Spearman's coefficient ρ_s.

S4 Effect of Population

**Development-Stage**	ΔAIC	ΔBIC	$r_{partial}$	p**-value**
All	-355.671	-352.08	0.578	0.0
Development	-76.579	-74.795	0.523	0.0
Early Growth	-33.155	-30.996	0.175	0.167
Mature	-232.705	-229.83	0.573	0.0
Transitional	-38.149	-36.781	0.447	0.016

Table S2: Multivariate analysis of the potential confounding effect of population at the entire network level and within each development-stage subgroup. An OLS regression was performed with the dependent variable log(BHI) and covariates log(PageRank) and log(Population) for all cities in each indicated subgroup. The ΔAIC and ΔBIC show the change in the AIC and BIC when including and omitting the variable log(PageRank) respectively, indicating that in all cases the linear model with log(PageRank) included was selected despite having more free parameters. Additionally, we show the corresponding partial correlation $r_{partial}$ and its p-value when conditioning on population, which in all cases except for the 'Early Growth' subgroup—which produces a much weaker association between BHI and PageRank in the first place, as discussed in the main text—indicates that PageRank and BHI are significantly correlated beyond their co-association with population. Taken altogether, these results demonstrate a limited confounding effect of population on the association between PageRank and BHI.

S5 Core-Periphery Structure

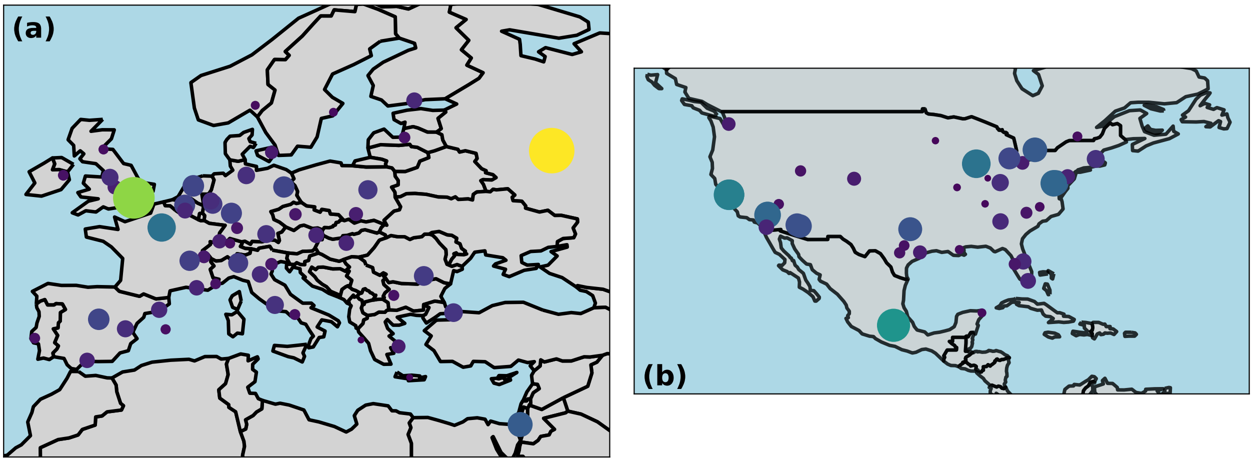

Figure S7: Two cores of the mobility network **a** coreness = 69 that consists mostly Western European cities, **b** coreness = 74 where the clusters are formed by North American cities. Size and colors of the nodes are proportional to their PageRank (from light to dark, large to small for high to low PageRank).

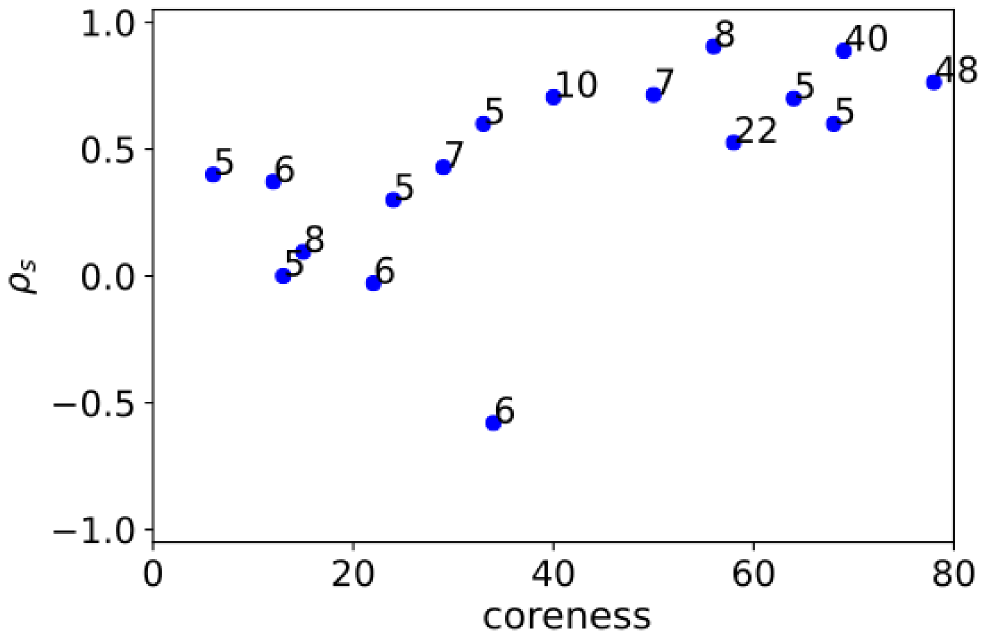

Figure S8: Correlation between BHI and PageRank (Spearman correlation coefficient is on the y-axis) in different clusters in the same *core* (having the same coreness value identified by $k-$core decomposition) where coreness is shown in the x-axis (from periphery to core left to right). $k-$core method identifies 2 dense clusters in the core of the mobility network with sizes 40 and 48, corresponding to North American and European cities as shown in Fig. S7 on the map. PageRank becomes less predictive for clusters of cities located in the outer layers of the mobility network. The numbers in each point represent the number of cities in each cluster.

S6 Exponent γ vs correlation coefficients

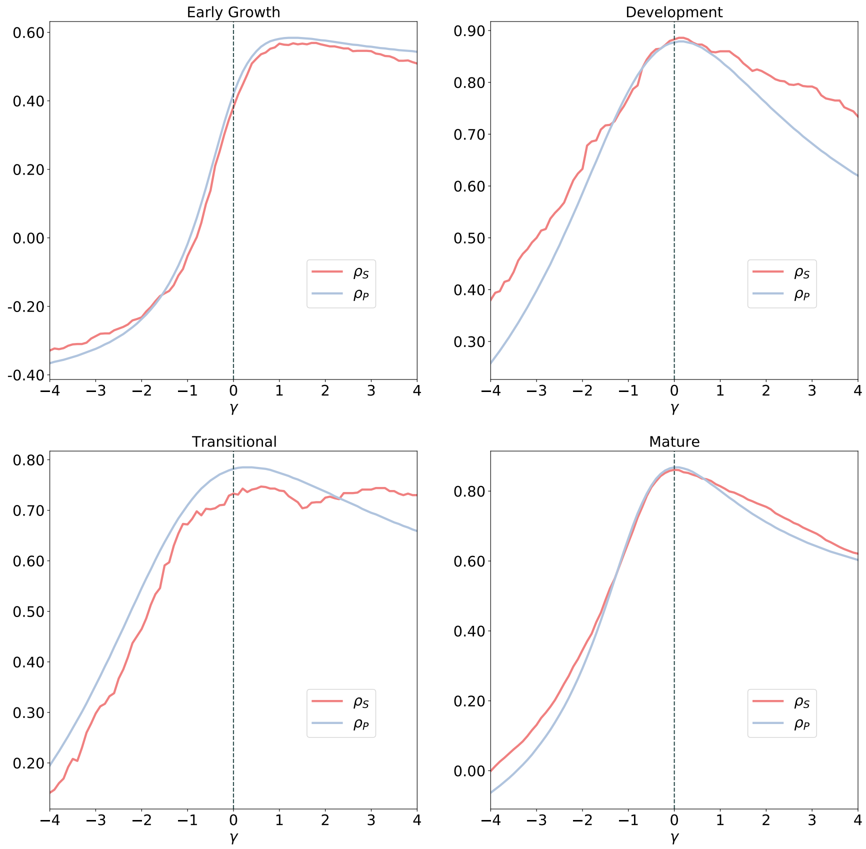

Figure S9: Dependence of the Spearman's correlation coefficient ρ_S (red) and the Pearson correlation coefficient ρ_P (blue) on the exponent γ (x-axis) governing how the success of the international connections of one city is relevant to shape its own success. The vertical dashed line represent the correlation existing when just accounting for the centrality of the city, measured by the PageRank, in the mobility network. The different categories correspond to different development stages of the cities.

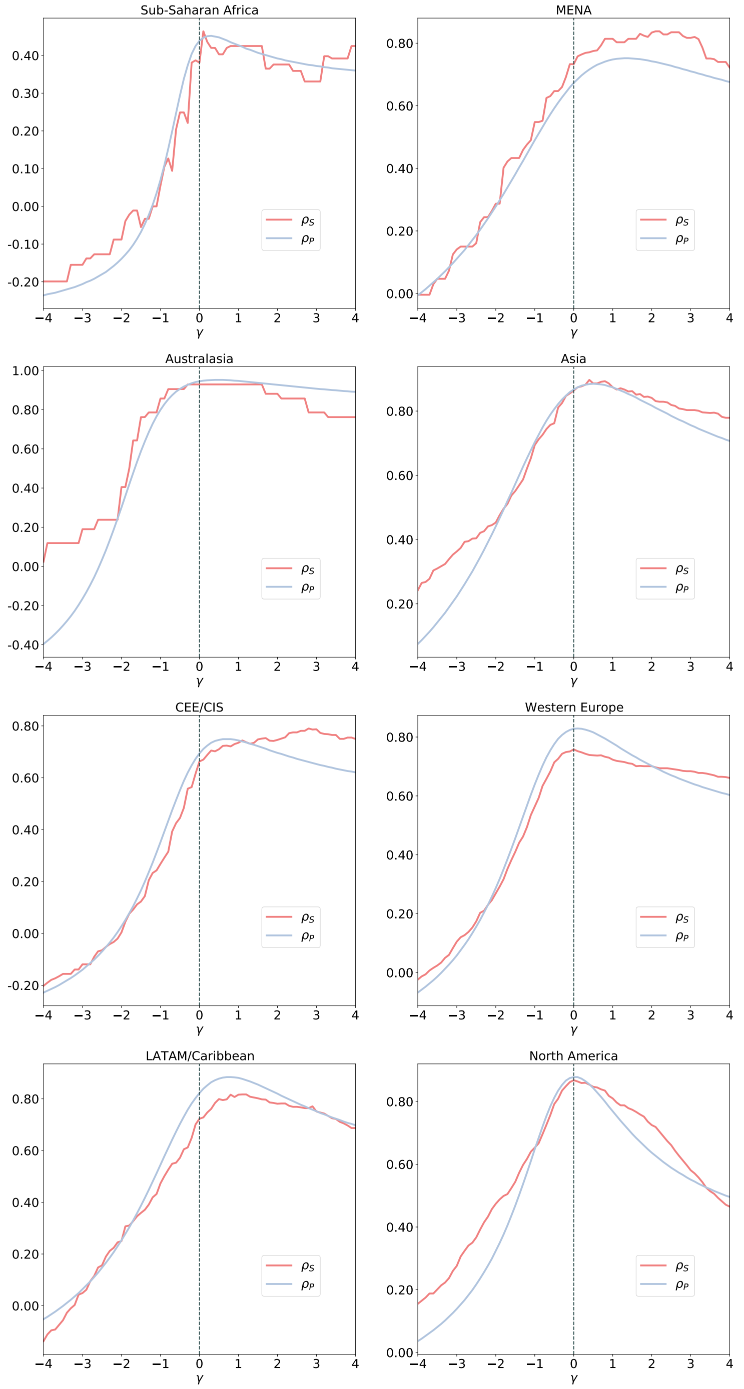

Figure S10: Dependence of the Spearman's correlation coefficient ρ_S (red) and the Pearson correlation coefficient ρ_P (blue) on the exponent γ (x-axis) governing how the success of the international connections of one city is relevant to shape its own success. The vertical dashed line represent the correlation existing when just accounting for the centrality of the city, measured by the PageRank, in the mobility network. The different categories contain cities located in different sub regions across the world.

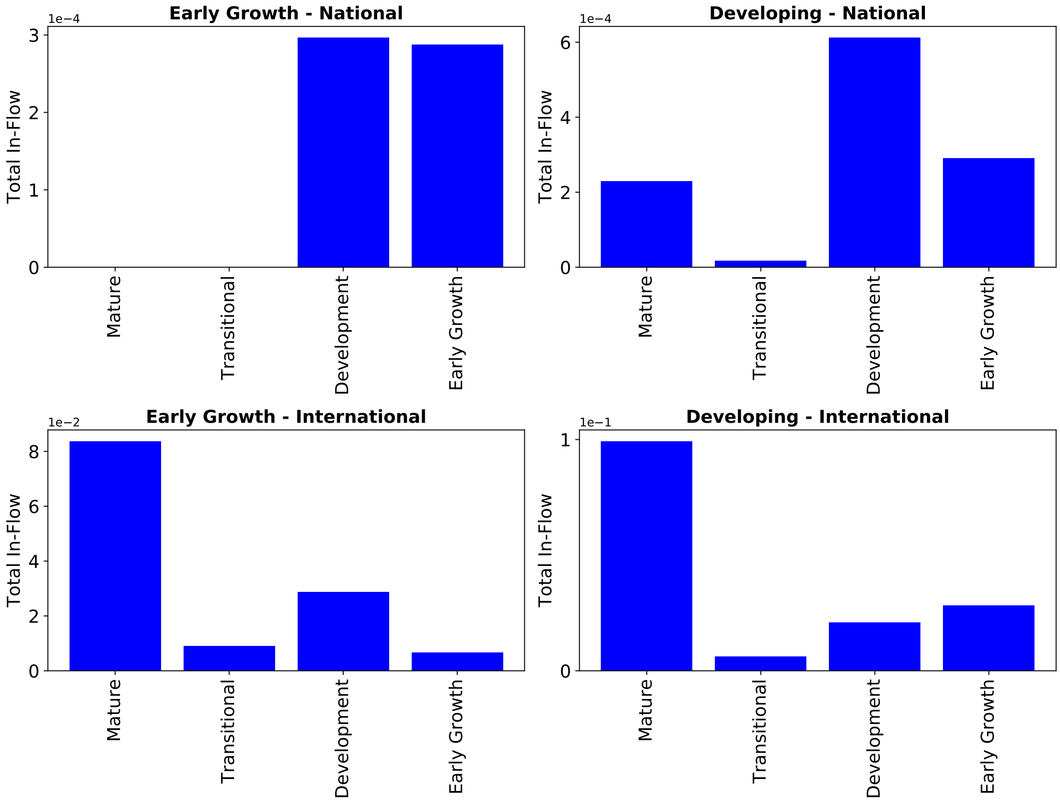

Figure S11: Breakdown of total in-flow of Early Growth and Developing cities, originating from national (upper panels) and international (lower panels) cities. International flows are dominated by Mature cities, which are insignificant in national inflows.

**Category**	**Classification**	**# of cities**	**# of flows**	$\hat{\mu}$	$\hat{\nu}$	r^2	ν_0	r_0^2	**Selected**	$\|\hat{\mu}/\hat{\nu}\|$
Development-Stage	Developing	44	1433	0.511	-1.838	0.963	-2.223	0.914	TRUE	0.278
Development-Stage	Early Growth	64	899	0.445	-3.078	0.908	-2.404	0.809	TRUE	0.144
Development-Stage	Mature	131	6395	0.206	-2.529	0.97	-2.705	0.969	TRUE	0.081
Development-Stage	Transitional	29	1032	0.163	-1.473	0.96	-1.365	0.955	TRUE	0.111
Region	North America	64	2903	0.188	-2.556	0.971	-2.739	0.97	TRUE	0.074
Region	LATAM and Caribbean	30	499	0.345	-1.377	0.942	-1.073	0.826	TRUE	0.251
Region	Asia	36	952	0.367	-1.643	0.95	-1.782	0.897	TRUE	0.223
Region	Australasia	8	148	0.44	-1.23	0.941	-0.634	0.719	TRUE	0.358
Region	Western Europe	69	3609	0.393	-2.492	0.778	-2.438	0.71	TRUE	0.158
Region	CEE/CIS	27	903	0.291	-1.847	0.402	-1.672	0.363	TRUE	0.157
Region	MENA	18	630	1.166	-9.011	0.946	-9.735	0.943	TRUE	0.129
Region	Sub-Saharan Africa	16	115	0.296	-1.851	0.999	-1.783	0.998	TRUE	0.16

Table S3: Results for flow model applied to both maturity and global sub-region city subgroups. The number of cities in each subgroup as well as the number of flows outgoing from these cities that were considered for the correlation analysis are shown. The inferred parameters from Eq. 8 are also shown, along with the corresponding coefficient of determination r^2. We additionally display the inferred parameter $\hat{\nu}_0$ for the restricted model where $\mu = 0$, as well as the corresponding coefficient of determination r_0^2. The column 'selected' denotes whether or not both the AIC and BIC difference between the two-parameter and one-parameter models are less than zero, indicating that the two-parameter model is preferred by these criteria. Finally, we show the ratio $|\hat{\mu}/\hat{\nu}|$, which indicates the relative importance of success versus distance in determining the flows for the corresponding subgroup.

**Category**	**Classification**	**# of cities**	**# of flows**	$\hat{\mu}$	$\hat{\nu}$	r^2	ν_0	r_0^2	**Selected**	$\lvert\hat{\mu}/\hat{\nu}\rvert$
Development-Stage	Developing	44	1433	1.048	-2.015	0.951	-2.223	0.914	TRUE	0.52
Development-Stage	Early Growth	64	899	0.908	-2.427	0.909	-2.404	0.809	FALSE	0.374
Development-Stage	Mature	131	6395	1.171	-3.015	0.981	-2.705	0.969	FALSE	0.389
Development-Stage	Transitional	29	1032	0.372	-1.437	0.962	-1.365	0.955	FALSE	0.259
Region	North America	64	2903	1.242	-3.115	0.982	-2.739	0.97	FALSE	0.399
Region	LATAM and Caribbean	30	499	0.795	-1.335	0.957	-1.073	0.826	FALSE	0.596
Region	Asia	36	952	0.916	-1.853	0.939	-1.782	0.897	TRUE	0.494
Region	Australasia	8	148	0.73	-1.254	0.901	-0.634	0.719	TRUE	0.582
Region	Western Europe	69	3609	0.705	-2.466	0.772	-2.438	0.71	FALSE	0.286
Region	CEE/CIS	27	903	1.904	-5.016	0.53	-1.672	0.363	FALSE	0.38
Region	MENA	18	630	1.905	-2.417	0.959	-9.734	0.943	FALSE	0.788
Region	Sub-Saharan Africa	16	115	0.634	-1.612	0.999	-1.783	0.998	FALSE	0.394

Table S4: Results for flow model applied to both maturity and global sub-region city subgroups, with GDP as the success measure S_j.
